# Factors associated with mortality in persons co-infected with tuberculosis and HIV in Suriname: a retrospective cohort study

**DOI:** 10.26633/RPSP.2019.103

**Published:** 2019-12-20

**Authors:** Deborah Stijnberg, Eric Commiesie, Diana Marín, Ward Schrooten, Freddy Perez, Mauro Sanchez

**Affiliations:** 1 Ministry of Health Ministry of Health Paramaribo Suriname Ministry of Health, Paramaribo, Suriname.; 2 National Tuberculosis Program National Tuberculosis Program Paramaribo Suriname National Tuberculosis Program, Paramaribo, Suriname.; 3 Universidad Pontificia Bolivariana Universidad Pontificia Bolivariana Medellín Colombia Universidad Pontificia Bolivariana, Medellín, Colombia.; 4 Hasselt University Hasselt University Hasselt Belgium Hasselt University, Hasselt, Belgium.; 5 Department of Communicable Diseases and Environmental Determinants of Health Pan American Health Organization/World Health Organization Washington, DC United States of America Department of Communicable Diseases and Environmental Determinants of Health, Pan American Health Organization/World Health Organization, Washington, DC, United States of America.; 6 Universidade de Brasilia Universidade de Brasilia Brasilia Brazil Universidade de Brasilia, Brasilia, Brazil

**Keywords:** Tuberculosis, HIV, mortality, National Health Programs, Suriname, Tuberculosis, VIH, mortalidad, programas nacionales de salud, Suriname, Tuberculose, HIV, mortalidade, programas nacionais de saúde, Suriname

## Abstract

**Objective.:**

To identify socio-demographic and clinical factors associated with mortality among persons with tuberculosis (TB) and TB/HIV co-infection in Suriname.

**Methods.:**

This was a retrospective cohort study using data from the national TB and HIV databases for 2010 – 2015. The survival probability of TB and TB/HIV co-infected patients was analyzed using the Kaplan-Meier estimates and the log-rank test. A Cox proportional hazard model was applied.

**Results.:**

The study showed that HIV-seropositivity (aHR: 2.08, 95%CI: 1.48 – 2.92) and older age (aHR: 5.84, 95%CI: 3.00 – 11.4) are statistically associated with higher mortality. For the TB/HIV co-infected patients, TB treatment (aHR: 0.43, 95%CI: 0.35 – 0.53) reduces the risk of death. Similarly, HIV treatment started within 56 days (aHR: 0.15, 95%CI: 0.12 – 0.19) and delayed (aHR: 0.25, 95%CI: 0.13 – 0.47) result in less hazard for mortality; Directly-Observed Treatment (aOR: 0.16, 95%CI: 0.09 – 0.29) further reduces the risk.

**Conclusions.:**

The Ministry of Health of Suriname should develop strategies for early case-finding in key populations, such as for HIV and TB in men 60 years of age and older. Implementation of Isoniazid Preventive Therapy for HIV should be pursued. Scaling up TB and HIV treatment, preferably through supervision, are essential to reducing the TB/HIV mortality.

## RESUMEN

**Objetivo.:**

Identificar factores sociodemográficos y clínicos asociados con la mortalidad en personas con tuberculosis (TB) y VIH en Suriname.

**Métodos.:**

Estudio de cohorte retrospectivo llevado a cabo con información de las bases de datos nacionales de TB y VIH para el período 2010-2015. Se analizó la probabilidad de supervivencia de los pacientes con TB y con coinfección TB/VIH mediante estimaciones de Kaplan-Meier y prueba de log-rank. Se aplicó un modelo de riesgo proporcional de Cox.

**Resultados.:**

El estudio demostró que la seropositividad al VIH (cociente de riesgos instantáneos ajustado [aHR]: 2,08, IC 95%: 1,48-2,92) y la edad avanzada (aHR: 5,84, IC 95%: 3,00-11,4) están estadísticamente asociados con una mayor mortalidad. En los pacientes coinfectados con TB/VIH, el tratamiento de la TB (aHR: 0,43, IC 95%: 0,35-0,53) disminuye el riesgo de muerte. Del mismo modo, el inicio dentro de 56 días del tra-tamiento antirretroviral (aHR: 0,15, IC 95%: 0,12-0,19) y retrasado (aHR: 0,25, IC 95%: 0,13-0,47) conllevan un menor riesgo de mortalidad; el tratamiento directamente observado (aOR: 0,16, IC 95%: 0,09-0,29) reduce aún más el riesgo.

**Conclusiones.:**

El Ministerio de Salud de Suriname debe desarrollar estrategias para la búsqueda temprana de casos de TB y VIH en poblaciones clave, como en los varones de 60 años de edad o mayores. Debería establecerse el tratamiento preventivo con isoniazida en las personas con VIH. A fin de reducir la mortalidad debida a la TB y el VIH es esencial ampliar el tratamiento de ambas enfermedades, preferiblemente de manera supervisada.

## RESUMO

**Objetivo.:**

Identificar fatores sociodemográficos e clínicos associados à mortalidade em pessoas com tuberculose (TB) e coinfecção pelo vírus da imunodeficiência humana (HIV) no Suriname.

**Métodos.:**

Foi realizado um estudo de coorte retrospectivo. As informações foram obtidas das bases de dados nacionais de TB e HIV para o período de 2010 a 2015. A probabilidade de sobrevida dos pacientes com coinfecção por TB/HIV foi analisada a partir de estimativas de Kaplan-Meier e pelo teste *log-rank*. Um modelo de riscos proporcionais de Cox foi aplicado.

**Resultados.:**

O estudo mostrou que soropositividade para o HIV (adjusted hazard ratio [aHR]: 2,08; IC95%: 1,48 a 2,92) e idade avançada (aHR: 5,84; IC95%: 3,00 a 11,4) foram estatisticamente associadas a maior mortalidade. Em pacientes coinfectados por TB e HIV, o tratamento da TB (aHR: 0,43; IC95%: 0,35 a 0,53) reduziu o risco de morte. Da mesma forma, o tratamento do HIV iniciado em 56 dias (aHR: 0,15; IC95%: 0,12 a 0,19) e retardado (aHR: 0,25; IC95%: 0,13 a 0,47) resultou em menor risco de mortalidade. O tratamento diretamente observado da tuberculose (aOR: 0,16; IC 95%: 0,09 a 0,29) reduziu ainda mais o risco.

**Conclusões.:**

O Ministério da Saúde do Suriname deve desenvolver estratégias para a detecção precoce de casos em populações-chave, tais como homens com 60 anos de idade ou mais. A implementação da terapia preventiva com isoniazida para o HIV deve ser mantida. A intensificação do tratamento de TB e HIV, preferencialmente através da supervisão, é essencial para reduzir a mortalidade por TB/HIV.

With an estimated 1.3 million deaths in 2016 among persons with tuberculosis (TB) and an additional 374 000 among TB patients co-infected with HIV, TB remains one of the top 10 causes of death in the world (1). Because the disease is a frequent and early opportunistic infection among HIV-infected patients, the TB and HIV epidemics are closely linked. HIV patients are 26 – 31 times more likely to develop TB, and both diseases are known to accelerate the other’s progression (2). Worldwide, 10% of TB patients are co-infected with HIV (1). In 2016, the global case-fatality rate among TB patients was 16%; targeted interventions are needed to achieve a 90% reduction by 2030, as set forth in the Sustainable Development Goals (1).

The Republic of Suriname is a Caribbean country located in northeastern South America. It has two urban, six rural, and two “interior” rural districts. The urban districts of Paramaribo (the capital) and Wanica cover only 0.5 % of the land surface, but contain 70% of the total population (3).

TB case finding in Suriname is passive. Diagnosis is by sputum smear microscopy and chest radiography. The National Tuberculosis Program (NTP), a department of the Ministry of Health, collaborates with primary health care organizations in the interior and coastal area for sputum collection in suspected cases and treatment follow up. Direct-Observation Treatment (DOT) workers are community members trained by the NTP to assist patients in taking their medication. All TB diagnostic services and treatments are free of charge. Testing all TB patients for HIV is the national policy.

HIV testing is widely available in Suriname. The country has adopted the public health approach wherein HIV-positive persons are referred to their primary care physician for a complete medical examination and laboratory testing (CD4 and Viral Load [VL]). At the time of the study, antiretroviral therapy (ART) was prescribed for patients who have a CD4 of ≤ 200 and a readiness to start treatment. For all patients, ART is available free of charge; for TB patients, ART is given in conjunction with TB medication, irrespective of CD4 level. Isoniazid Preventive Therapy is given to the contacts of TB patients following a positive tuberculin skin test. It is not routinely given to HIV patients.

In 2010 – 2015, 95% of TB patients in Suriname were tested for HIV and 25% – 30% were found to be HIV positive. A high mortality of 10% – 15% has been reported annually among TB patients (4). A previous study in Suriname found a 5-fold increase in mortality among the TB/HIV co-infected in 2010 – 2013 (*P* < 0.05), after adjusting for diabetes mellitus (DM) which was also found to be significantly correlated with mortality (4).

A study in Europe and Latin America in 2011 – 2013 found that TB-related mortality among HIV-patients was associated with factors such as suboptimal initial TB treatment due to lack of drug susceptibility and low CD4 cell count (5). Additionally, a meta-analysis showed that ART before or during TB treatment reduced the risk of less favorable outcomes; however, early immunological failure despite viral suppression was still found to be associated with mortality after ART initiation in patients with advanced TB/HIV patients (6). A retrospective cohort study undertaken in an acute-care hospital in Hong Kong, identified factors such as difficulties in diagnosis, non-HIV immune suppression, respiratory failure, and advanced age to be associated with mortality among TB patients in a low HIV prevalence setting (7). In Latin America and the Caribbean, Multi-Drug Resistant TB and indigenous ethnicity were associated with mortality in TB patients, while availability of water and basic sanitation, literacy of women, and good nutritional status reduced mortality rates (8). There is limited information on the factors that influence high mortality among TB patients in Suriname.

The objective of this study was to identify socio-demographic and clinical factors associated with mortality among a cohort of persons with TB and TB/HIV in Suriname.

## MATERIALS AND METHODS

This was a retrospective cohort study based on routinely-collected data on TB cases in Suriname, notified from January 2010 – December 2015. The TB/HIV co-infected patients within the selected cohort were also analyzed separately. No sample size was calculated because the study examined the entire national TB-notified population for that period.

### Data collection

Data was collected from two databases: the National TB Database and the National HIV Patient Master Index. The National TB Database is maintained by the NTP and collects demographic and clinical information on notified TB cases, as well as programmatic variables such patient enrollment in DOT. It is updated continuously according to active surveillance by TB laboratories nationwide. The National HIV Patient Master Index is established through probabilistic matching (fuzzy matching) and uses a unique identifier to link HIV testing and treatment, CD4/VL, and the national Prevention-Mother-To-Child-Transmission database. The HIV Patient Master Index contains the deduplicated unique patient cohort. The NTP was linked to it to extract relevant information from the HIV Master Index regarding TB/HIV co-infected patients. For persons registered as HIV positive in the TB database, but not found in the HIV Master Index, the matched unduplicated code was identified. This was then used to extract information from the individual HIV sources instead of the deduplicated HIV Master Index. For all the unique identifiers that were linked, relevant clinical information, such as HIV enrollment date and ART initiation, was extracted and added to the TB database.

### Variables

The main outcome was mortality. The enrollment duration—defined as TB diagnosis until either treatment completion, death, or lost to follow-up—was considered.

The following independent factors were investigated: sex; age; ethnicity; type of TB case (new/ retreatment); TB location (pulmonary or outside the lungs, i.e., extra-pulmonary); TB treatment under supervision (DOT, yes/no); having received TB treatment (yes/no); and having been hospitalized for ≥ 1 day during the current TB episode ( yes/no admittance). “Having received TB treatment” included treatment based on positive smears and empirically treated patients (based on clinical suspicion). “No TB treatment” included people diagnosed with TB who died prior to starting treatment and those lost to follow-up. The extra-pulmonary cases were diagnosed based on histology or clinical suspicion (9).

The HIV-specific independent variables used the CD4 count closest to the TB enrollment date: < 50, 51 – 200, 201 – 350, or ≥ 350 cells/μl. Antiretroviral therapy was either “no ART,” “early,” (initiation of ART within ≤ 56 days of TB diagnosis), or “delayed” (≥ 57 days).

### Statistical analyses

Patient description and mortality calculation were done according to socio-demographic and clinical characteristics.

Survival was estimated with the Kaplan-Meier method and differences evaluated with the log-rank test. Hazard Ratios (HR) and 95% confidence intervals (95%CI) were calculated using Cox regression with standard errors adjusted by living area (urban, rural, and interior) to identify the factors associated with mortality in bivariate and multivariate models.

In the multivariate models, the variables were tested with a *P* < 0.25. Variables included in the TB model were sex, age, TB location, hospitalization, DOT, and TB treatment. For the TB/HIV model, the variables considered were: case type, DOT, TB treatment, ART use, and CD4 category at TB diagnosis. A two-tailed *P* value < 0.05 was considered significant. The proportional hazards assumption was checked based on Schoenfeld residuals. Analyses were performed using Stata® 11.1 (StataCorp, College Station, Texas, United States).

### Ethics

The Ministry of Health of Suriname approved the study following a review by the *Commissie Mensgebonden Wetenschappelijk Onderzoek*, which is the national ethics committee. Ethical clearance was also received from the PAHO Ethics Review Committee. Since this was a retrospective study using routinely-collected surveillance data, informed consent was not needed. Confidentiality was guaranteed by using only the unique identifiers.

## RESULTS

From 2010 – 2015, a total of 917 cases of TB, including 252 TB/HIV co-infected cases, were notified to the NTP of Suriname. All TB cases were included in the study.

### TB cohort

Of the total TB patients, 84% (769/917) were 15 – 59 years, with a median age of 41 years (interquartile range [IQR]: 31 – 52). Most were male (71%), new cases (91%), had pulmonary TB (87%), and were admitted to the hospital (82%). Median duration of hospital stays was 29 days (IQR: 10 – 53). TB treatment was provided to 93% of cases, with 50% receiving DOT, which led to a treatment success rate of 70%.

**FIGURE 1.**
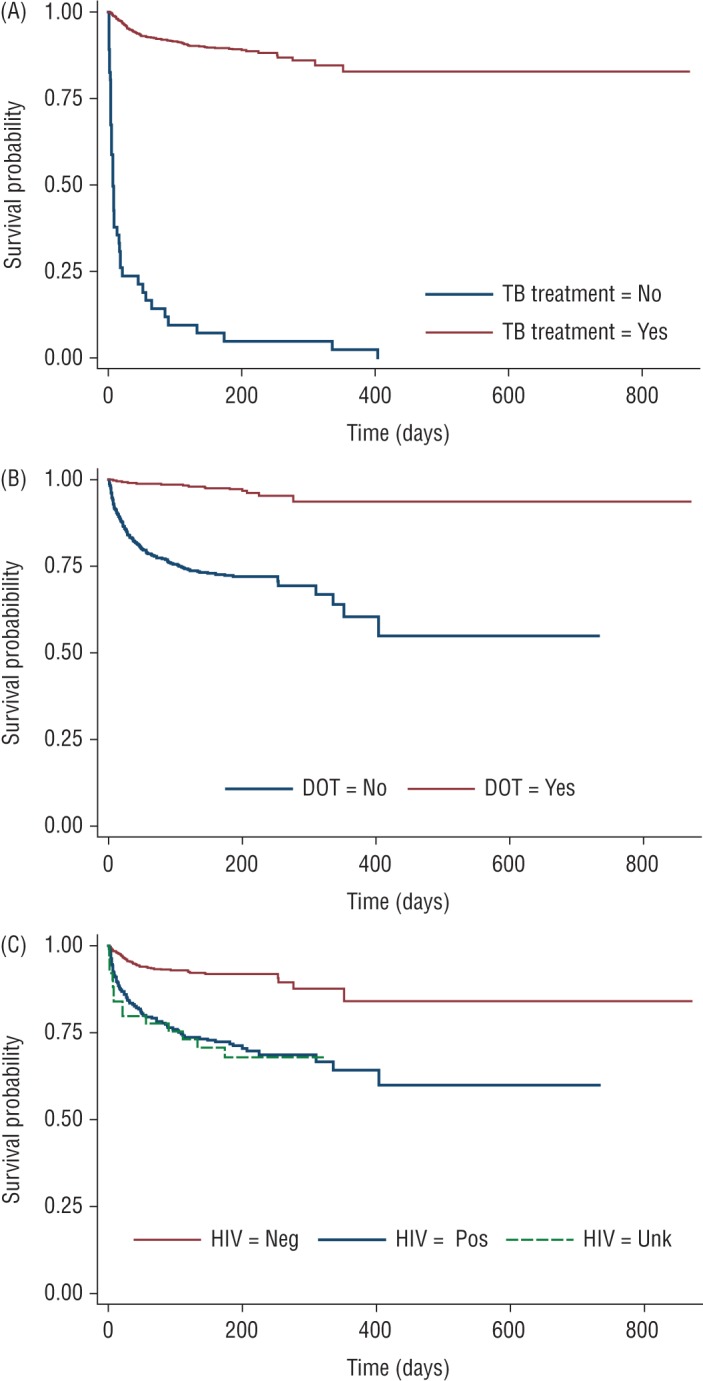
Effect of Directly-Observed Treatment (DOT), tuberculosis (TB) treatment, and HIV serostatus on the probability of survival in TB notified cases in Suriname, 2010 – 2015

***Note:*** The inset shows Kaplan-Meier survival estimates according to DOT (panel A), having received TB medication (panel B), and HIV serostatus—Neg: Negative, Pos: Positive, and Unk: Unknown (panel C).

***Source:*** Prepared by the authors from the study results.

Overall, the mortality rate was 16% (95%CI: 13.4 – 18.1). A higher mortality rate was observed in the creole ethnic group, the hospitalized, not having obtained DOT, not having received TB treatment, and those with a positive or unknown HIV status (Table 1). Applying Kaplan-Meier estimations, the survival was lower in patients not receiving DOT for TB and being HIV- infected (Figure 1).

The risk factors associated with higher mortality in TB cases were being male, 60 years of age or older, extra-pulmonary tuberculosis, having been hospitalized, not having received DOT, and being HIV positive (Table 1).

### TB/HIV subgroup analyses

For the 252 TB/HIV co-infected patients, the median duration of enrollment for a TB episode was 186 days (IQR: 50 – 239). The median age was 42 years (IQR: 35 – 50), similar to the TB cohort. After linking with the National HIV Patient Master Index, we found that 80% of TB/HIV patients were diagnosed with HIV a median of 521 days (IQR: 5 – 1 731) before enrollment for TB.

Among this subgroup, 94% of patients (236/252) were admitted to the hospital, with 50% staying for 32 days (IQR: 13 – 56) and 88% receiving TB treatment. Based on the treatment information from the HIV Patient Master Index, 73% received treatment for both HIV and TB. Overall, the mortality ratio was 29% (95%CI: 24 – 35). The mortality rate among the cases receiving empirical treatment, meaning treatment without a bacteriological confirmatory test, compared to the confirmed cases was respectively 43% versus 22%.

**FIGURE 2.**
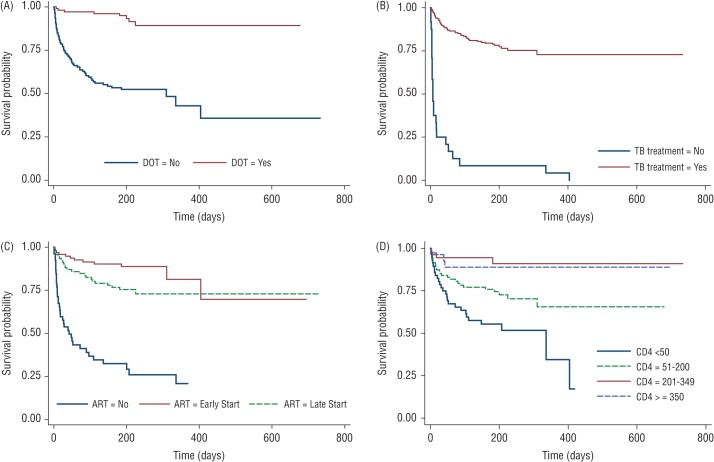
Effect of Directly-Observed Treatment (DOT), tuberculosis (TB) treatment, antiretroviral use (ART), and CD4 at TB diagnosis on the probability of survival in TB/HIV notified cases in Suriname, 2010 – 2015

***Note:*** The inset shows Kaplan-Meier survival estimates according to DOT and having received TB medication (panels A and B). The time of ART initiation compared to start TB treatment and the CD4 at TB diagnosis in cells/μl are shown in panels C and D.

***Source:*** Prepared by the authors from the study results

Co-relating the mortality percentage among the different demographic and clinical features showed that being hospitalized, receiving DOT, taking TB treatment, and having started ART are related to a lower mortality percentage (Table 2). In the multivariate model, the risk factors associated with lower mortality in TB/HIV patients were retreatment cases, receiving TB treatment, DOT, and using ART whether having started early or delayed. Furthermore, unknown TB case type and having a CD4 < 350 at TB diagnosis were significantly associated with higher mortality (Table 2 and Figure 2).

**TABLE 1.** Demographic and clinical features associated with mortality among tuberculosis (TB) notified cases in Suriname, 2010 – 2015

Characteristic	Total (*n*= 917)	Mortality (*n* = 143)		*P* value^a^	HR crude (95%CI)^b^	HR adjusted (95%CI)^c^
							n	%			
Sex				0.206		
Male	648	108	16.7		1	1
Female	269	35	13.0		0.81 (0.70 - 0.95)	0.88 (0.85 - 0.92)^d^
Age group (years)				< 0.001		
< 15	48	6	12.5		1	1
15 – 34	268	19	7.1		0.56 (0.34 - 0.90)	1.01 (0.36 - 2.85)
35 – 49	335	58	17.3		1.25 (0.71 - 2.18)	1.86 (0.58 - 6.00)
50 – 59	166	35	21.1		1.62 (0.86 - 3.06)	2.22 (0.90 - 5.49)
≥ 60	100	25	25.0		1.79 (0.95 - 3.38)	5.84 (3.00 - 11.4)^d^
Ethnicity^e^				0.649		
Creole	360	60	16.7		1.68 (0.65 - 4.31)	
Maroon	130	16	12.3		1.28 (0.35 - 4.64)	
Hindustani	130	25	19.2		1.95 (0.59 - 6.49)	
Mixed	110	18	16.4		1.73 (1.17 - 2.56)	
Javanese	91	10	11.0		1	
Amer-Indian	76	11	14.5		1.36 (0.30 - 6.11)	
Other / Unknown	20	3	15.0		1.52 (0.13 - 17.7)	
TB case type				0.59		
New	834	129	15.5		1	
Retreatment	76	12	15.8		0.84 (0.74 - 0.95)	
Unknown	7	2	28.6		2.20 (1.82 - 2.64)	
TB location				0.246		
Pulmonary	799	117	14.6		0.73 (0.67 - 0.79)	0.63 (0.56 - 0.72)^d^
Extra-pulmonary	116	24	20.7		1	1
Hospitalization				0.004		
Yes	753	129	17.1		2.58 (1.64 - 4.06)	5.92 (4.23 - 8.29)^d^
No	164	14	8.5		1	1
Directly-Observed Treatment				< 0.001		
Yes	449	16	3.6		0.10 (0.07 - 0.13)	0.13 (0.10 - 0.17)^d^
No	468	127	27.1		1	1
TB treatment				< 0.001		
Yes	848	95	11.2		0.04 (0.03 - 0.04)	0.04 (0.04 - 0.06)^d^
No	69	48	69.6		1	1
Diabetes mellitus				0.696		
Yes	87	15	17.2		1.21 (0.84 - 1.75)	
No	830	128	15.4		1	
HIV				< 0.001		
Positive	252	74	29.4		3.58 (2.74 - 4.69)	2.08 (1.48 - 2.92)^d^
Negative	603	51	8.5		1	1
Unknown	62	18	29.0		4.25 (3.38 - 5.36)	1.05 (0.27 - 4.14)

^a^ Log-rank test.

^b^ Hazard Ratio crude estimated with Cox regression and standard errors adjusted by living area (urban, rural, and rural interior).

^c^ Hazard Ratio adjusted by sex, age, TB location, hospitalization, DOT, TB treatment, and HIV infection. Standard errors adjusted by living area (urban, rural, and rural interior).

^d^ *P* < 0.01

^e^ The Maroon are people of African descent; the Creoles are African and other ethnicities, mostly European; Hindustani are descendants of India; and the Javanese are originally from Java.

***Source:*** Prepared by the authors from the study results.

## DISCUSSION

Based on data from 917 people diagnosed with TB in Suriname from 2010 – 2015, this study found that HIV co-infection has an adjusted 2.08 higher risk of mortality. Similarly, older age, males, and hospitalized patients were at higher risk of death. In line with international recommendations, receiving TB and HIV treatment, especially when DOT was applied, reduced the risk of mortality significantly in both TB and TB/HIV co-infected patients.

Similar to findings by Commiesie in 2013 (4), after adjusting for other factors, HIV-infection is a strong predictor for mortality. Various studies have shown similar findings. In Brazil, a 40% increase in non-TB causes of death was found among HIV-infected developing active TB (10). Early immunologic response was the explanation given by Ravimohan and colleagues (11) for early mortality among TB/HIV co-infected patients with advanced HIV and active TB. The high prevalence of HIV (29.5%) among the TB notified cases in Suriname in 2010 – 2015 is noteworthy given how much higher it is than the global prevalence of 10% (1). Possible explanations could be the inconsistent implementation of IPT for people living with HIV and the initiation of treatment with CD4 200. As highlighted in the TEMPRANO study (12), IPT reduces the incidence of active TB by 60% (aHR 0.44, 95%CI: 0.28 – 0.69) and early initiation of ART reduces it by one-half (aHR 0.50, 95%CI: 0.32 – 0.79). The higher mortality rate among those clinically-diagnosed than those bacteriologically confirmed is similar to findings by Adamu and colleagues (13) who observed it to be almost 5 times higher (aHR 4.96, 95%CI: 2.69 – 9.17). Their possible explanations were poor access to diagnostic testing and misdiagnosis of other diseases, especially HIV and extra-pulmonary TB (13).

**TABLE 2.** Demographic and clinical features associated with mortality among TB/HIV notified cases in Suriname, 2010 – 2015

Characteristic	Total (*n* = 252)	Mortality (*n* = 74)	*P* value^a^	HR crude (95%CI)^b^	HR adjusted (95%CI)^c^
			n	%			
Sex				0.356		
Male	172	54	31.4		1	
Female	80	20	25.0		0.81 (0.64 - 1.03)	
Age group (years)				0.181		
< 35	65	14	21.5		1	
35 – 49	121	37	30.6		1.30 (1.07 - 1.57)	
50 – 59	57	18	31.6		1.41 (0.85 - 2.32)	
≥ 60	9	5	55.6		2.52 (1.35 - 4.67)	
Ethnicity^d^				0.285		
Creole	143	40	28.0		1.51 (0.40 - 5.66)	
Maroon	39	8	20.5		1	
Hindustani	28	12	42.9		2.14 (0.56 - 8.17)	
Mixed	31	9	29.0		1.56 (0.29 - 8.44)	
Other / Unknown	11	5	45.5		2.94 (0.98 - 8.77)	
TB case type				0.228		
New	211	64	30.3		1	1
Retreatment	37	8	21.6		0.47 (0.26 - 0.84)	0.64 (0.45 - 0.91)^e^
Unknown	4	2	50.0		1.84 (1.39 - 2.44)	5.13 (4.67 - 5.64)^f^
TB location				0.956		
Pulmonary	207	58	28.0		0.92 (0.65 - 1.30)	
Extra-pulmonary	45	16	35.6		1	
Hospitalization				0.396		
Yes	236	67	28.4		0.66 (0.50 - 0.89)	
No	16	7	43.8		1	
Directly-Observed Treatment				< 0.001		
Yes	105	8	7.6		0.12 (0.09 - 0.16)	0.16 (0.09 - 0.29)^f^
No	147	66	44.9		1	1
TB treatment				< 0.001		
Yes	221	49	22.2		0.08 (0.06 - 0.10)	0.43 (0.35 - 0.53)^f^
No	31	25	80.6		1	1
Diabetes mellitus				0.56		
Yes	1	0	0.0		-	
No	251	74	29.5			
Antiretroviral Therapy				< 0.001		
No	54	38	70.4		1	1
Early start	102	12	11.8		0.11 (0.09 - 0.14)	0.15 (0.12 - 0.19)^f^
Late start	94	23	24.5		0.22 (0.12 - 0.38)	0.25 (0.13 - 0.47)^f^
CD4 category^g^				< 0.001		
< 50	58	27	46.6		16.8 (12.0 - 23.6)	5.83 (3.02 - 11.2)^f^
51 – 200	99	27	27.3		7.43 (4.24 - 13.1)	5.49 (3.28 - 9.20)^f^
201 – 349	38	3	7.9		2.25 (1.58 - 3.22)	1.30 (1.17 - 1.45)^f^
≥ 350	28	3	10.7		1	1

^a^ Log-rank test.

^b^ Hazard Ratio crude estimated with Cox regression and standard errors adjusted by living area (urban, rural, and rural interior).

^c^ Hazard Ratio adjusted by TB case type, DOT, TB treatment, ART use, and CD4 category at TB diagnosis. Standard errors adjusted by living area (urban, rural, and rural interior).

^d^ The Maroon are people of African descent; the Creoles are African and other ethnicities, mostly European; Hindustani are descendants of India; and the Javanese are originally from Java

^e^ *P* < 0.05

^f^ *P* < 0.01

^g^ CD4 at TB diagnosis in cells/μl.

***Source:*** Prepared by the authors from the study results.

Contrary to Commiesie (4), DM was not associated with mortality. A possible explanation is the role of HIV, since 30% of people in our study without DM were HIV positive compared to 1% of diabetic patients. Previously, the association between DM and mortality was found by excluding HIV from the analysis. In Tanzania, in the first 100 days of TB treatment, a risk of death 2 – 5 time higher was found among HIV uninfected diabetic TB patients (14).

Several studies indicate that older age, being male, pulmonary TB, and TB treatment are associated with mortality among TB patients (8, 16). Additionally, non-HIV immuno-compromising factors, such as chronic kidney disease, malignancies, and alcohol/drug abuse, were correlated with mortality among TB patients (8, 16, 17). As found in our study, previous reports showed a positive effect of treatment on TB and HIV. In India (17), higher mortality was associated with not using ART (aOR: 2.8, 95%CI: 1.15 – 6.81) or cotrimoxazole (aOR: 3.46, 95%CI: 1.47 – 8.14). In Suriname, the guideline at that time of a study (9) encouraged starting cotrimoxazole for all co-infected patients, but treatment could be deferred with CD4 > 200. In the present study, data on cotrimoxazole use was not included because it was not being reliably and consistently collected nationally during the study period.

A meta-analysis (6) found a 44% – 71% mortality reduction with ART initiation (Relative Risk = 0.42, 95%CI: 0.29 – 0.56). A study from South Africa (18) found a 56% relative risk reduction of mortality when TB and HIV therapy were provided in conjunction, rather than giving ART after completing TB treatment (HR: 0.44; 95%CI: 0.25 – 0.79). Moreover, it is recommended that ART be started within 8 weeks of initiating TB treatment or within 2 weeks for those with CD4 < 50 (20, 21). Suriname’s treatment guidelines align with this recommendation, i.e., ART should be initiated 2 – 8 weeks into TB treatment (9). A recent report from Malaysia indicates that in addition to not using ART and a CD4 ≤ 200 (aHR: 3.89, 95%CI: 1.20 – 12.63), having three or more opportunistic infections (aHR: 3.61, 95%CI: 1.04 – 12.53) are risk factors for mortality in TB/HIV co-infected individuals (21).

A study in Suriname (22) showed that people successfully treated in 2010 – 2015 were 6.4 times more likely to be enrolled in DOT (*P* < 0.05); this could explain the effect of DOT. A surprising finding in our study was the reduced risk of mortality among the retreatment cases. In addition, Vijay and colleagues found a univariate relation between retreatment cases and mortality, which they related to a reduction in drug susceptibility (17). The absence of association in our study could be explained by the fact that drug resistance in Suriname is rare (23) and the sample of retreatment cases was small.

This study had strengths worth noting. Matching the National TB Database with the National HIV Patient Master Index produced complementary and more complete data than previously obtained, despite some inconsistencies that required data cleaning. The large sample size—all notified cases in Suriname from 2010 – 2015—and data on time of enrollment were also strengths of this study.

### Limitations

A limitation of this study was that it did not consider other possible risk factors, such as malignancies, alcohol and drug abuse, and smoking (both active and passive). Evidence shows that smoking is an important factor in increased TB morbidity and mortality and reduced effectiveness of ART (24). Additionally, other social factors, such as literacy and sanitation, were not considered and have proven to be important in Latin America and the Caribbean (8). Furthermore, mortality among patients lost to follow-up was unknown. Lastly, the influence of drug resistance was not considered, however it is unlikely to have been important since resistance to rifampicin was shown to be low in Suriname (23).

### Conclusions

The associations found between demographic and clinical factors and mortality in TB/HIV co-infected patients in Suriname highlight the importance of early initiation of TB treatment and ART. Supervised treatment adherence should be a priority, especially for males, persons 60 years of age and older, those with extra-pulmonary TB infection, and those TB/HIV co-infected with a CD4 count of ≤ 350.

Late diagnoses of HIV and TB also present a challenge. At HIV diagnosis, 50% had a CD4 ≤ 200 and 60% of HIV/TB co-infected patients waited ≥ 3 months for TB notification. These findings support the strategy proposed by the National AIDS Program of Suriname to increase early HIV case finding and initiate ART sooner to prevent TB. Suriname should also consider a protocol for Isoniazid Preventive Treatment, as recommended by WHO (25).

The findings of this study reinforce strict adherence to the national treatment protocol—starting TB treatment as soon as possible, preferably supervised (DOT), with ART following in 2 – 8 weeks for those with a TB/HIV co-infection (9). The Ministry of Health of Suriname should consider revising the protocol to include interventions for patients with different risk profiles.

In addition, the TB data collection process and its link to the HIV program data should be improved and performed routinely. Further research into risk and social factors, especially those not considered here, should be undertaken in the near future.

## Author contributions.

DS, EC, DM, and MS designed the study. DS, DM, and MS conducted the analyses with input from all authors. DS drafted the manuscript and all authors critically revised it. The final manuscript was reviewed and approved by all authors.

## Acknowledgements.

The authors would like to thank all SORT-IT Suriname trainers for their patience, knowledge, and valuable time, especially Manuel Sanchez who assisted in the preliminary phase of the protocol development. We also thank PAHO for providing the SORT-IT training, and all the colleagues who participated. We are also grateful for the TB and HIV program staff who keep the data updated. A special thanks to Mike McKee for updating the HIV master regularly and for his assistance with linking the TB data and the HIV national surveillance data.

## Disclaimer.

Authors hold sole responsibility for the views expressed in the manuscript, which may not necessarily reflect the opinion or policy of the *RPSP/PAJPH.*
